# Theory of Event Coding (TEC) V2.0: Representing and controlling perception and action

**DOI:** 10.3758/s13414-019-01779-4

**Published:** 2019-06-05

**Authors:** Bernhard Hommel

**Affiliations:** Institute of Psychology, Cognitive Psychology Unit, University of Leiden, Wassenaarseweg 52, 2333AK Leiden, The Netherlands

**Keywords:** Perception and action, Cognitive and attentional control

## Abstract

This article provides an update of the Theory of Event Coding (TEC), which claims that perception and action are identical processes operating on the same codes – event files consisting of integrated networks of sensorimotor feature codes. The original version of the theory emphasized its representational underpinnings, but recent theoretical developments provide the basis for a more integrated view consisting of both the codes that are shared between perception and action in the control processes operating on these codes. Four developments are discussed in more detail: The degree to which the integration and retrieval of event files depends on current goals, how metacontrol states impact the handling of event files, how feature binding relates to event learning, and how the integration of non-social events relates to the integration of social events. Case examples using various versions of the Simon task are used to explain how the new version of TEC explains interactions between perception and action in non-social and social situations.

## Introduction

The exact starting points of research agendas are notoriously difficult to determine, but it is fair to say that the 1960s were particularly important for the systematic experimental investigation of interactions between perception and action. To a substantial degree, research during that period was driven by applied interests in the construction and evaluation of ergonomic man-machine interfaces. Workers often had to respond to visual signals indicating the presence of some important state of affairs, such as the fuel level of a particular apparatus, by performing appropriate actions, such as initiating a refuel, and the question was how the signal should be presented in order to optimize the speed and accuracy of the response. Experiments were often theory-free and mainly played through various combinations of stimulus displays and response handles to determine the combination working best (e.g., Loveless, 1962). It quickly turned out that performance benefited from various kinds of feature overlap between stimulus display and response handle – an observation that was also made in various laboratory tasks, such as with the Stroop or the Simon task.

Unfortunately, however, the preferred kind of theorizing in the 1960s and 1970s provided only limited insight into the underlying cognitive principles. According to the dominating Sternberg logic at that time, a good cognitive theory was supposed to identify the processes that are relevant in producing a particular experimental effect and determine their ordering in time by assessing the temporal demands of the respective processes and their sensitivity to particular experimental manipulations (Sternberg, 1969). Applying this logic revealed that the effect of feature overlap between stimulus and response mainly affected the stage of response selection, in the sense that feature overlap reduces the time needed to select a response. Why that might be the case remained a mystery. For instance, one of the most comprehensive models to account for effects of stimulus-response feature overlap, the *Dimensional Overlap Model* of Kornblum, Hasbroucq, and Osman (1990), claimed that such feature overlap would automatically activate the respective response, which would be beneficial if this response is correct but impair performance if it is not. Given the process-oriented nature of the approach, it failed to explain *how* and *why* a stimulus would tend to activate feature-overlapping responses in the first place or which features of the response would be sensitive to overlap. It simply assumed that overlap is sufficient for automatic activation, which – given that evidence for the automatic activation of feature-overlapping responses was the explanandum the theory sought to explain – provided little more than a rewording of the original observation.

It was the limited scope of process-oriented approaches and their limited interest in truly mechanistic explanations of interactions between perception and action that motivated my colleagues and me to develop the *Theory of Event Coding* (TEC; Hommel, Müsseler, Aschersleben, & Prinz, 2001a). We felt that theorizing about processes without considering the codes on which such processes are operating, their format and origin, and their functional role in representing and processing perceived and produced events, is unlikely to generate deeper insights into the connection between perception and action. As a result of this dissatisfaction with purely process-oriented boxologies, TEC was very heavy on the representational side while making only a few assumptions with respect to processes. In particular, the original version of TEC did not provide much information about how the hypothesized representational codes interact with control processes, which was criticized by various commentators of the original target article and acknowledged in our reply (Hommel et al., 2001b; R2.7). To address this shortcoming, the recent developments of TEC were mainly aimed to integrate the representational assumptions with a functional control structure, i.e., to make derivations and predictions more specific and better testable, but also to further broaden the scope of the theory. It is these recent developments that I would like to focus on here, after having briefly presented the main assumptions of the original TEC (for broader theoretical justification and empirical basis I refer the interested reader to Hommel et al., 2001a, and Hommel, 2009, 2015a, 2016), which the new version leaves fully intact. In particular, I discuss how event coding is affected by control and metacontrol states and processes, how feature integration/binding relates to learning, and how the theory might be extended to social events. To provide a more detailed insight into how representational and control processes might be integrated, I then apply old and new theoretical developments to experimental effects, where I take variants of the well-known Simon task as a case example, before concluding with a few desiderata.

## The Theory of Event Coding V1.0

### General assumptions

TEC relies on three general and five more specific assumptions. In general terms, the theory assumes that (a) perceptions and action goals are coded in the same way (common coding), (b) through distributed feature codes, which (c) refer to the distal features of the represented event. The first of these assumptions derives from the ideomotor heritage of TEC. Ideomotor approaches intend to explain how people are able to perform goal-directed movements without having direct conscious access to or any conscious knowledge about their own motor system (Shin, Proctor, & Capaldi, 2010; Stock & Stock, 2004). The ideomotor principle that is thought to provide this explanation claims that carrying out movements is accompanied by a learning process that integrates the motor patterns driving the movement with the sensory information that the movement generates, such as the proprioceptive experience of moving and the visual changes of hand position, or the kinesthetic and auditory effects of touching a piano key. Note that part of the integrated re-afferent information refers to the movement itself, i.e., the bodily experience, while another part refers to the way the movement changes the environment. The integration of motor patterns and codes of re-afferent action effects renders the latter effective primes of the former, so that an agent can simply reactivate the action-effect codes (a process that different approaches have considered to consist of “thinking of,” “imagining,” or “simulating” particular action outcomes), which then tend to reactivate the motor patterns they are integrated with. It is through these action-effect codes that people gain access to their motor system, so that actions can be considered to be represented by codes of the sensory effects they have been experienced to produce. Note that the active use of such past knowledge for the control of future action can be taken to turn the knowledge into a prediction (or at least some kind of expectation that the previously produced action effect will again be produced by carrying out the action), which renders the ideomotor approach functionally equivalent to present predictive-coding models (Kilner et al., 2015).

The idea that actions are represented by codes of their perceptual effects begs the question in which sense perception and action actually differ in nature. Outside the psychological laboratory, perceived events are commonly actively produced by performing particular movements. This is obvious for touch, which requires the systematic movement of touch sensors across a surface with to-be-identified features, but holds for all sensory modalities, as we for instance foveate objects only because we have moved our body close to and directed our eyes at them. Hence, almost all perceptions can be considered effects of some preceding action, and the resulting percept often considers the characteristics of this action, as for instance the direction in which the eyes were moved in order to foveate the object – which determines the object’s location. If so, perception and action can be considered two processes that are not just related or interconnected but actually identical: The carrying out of some movement to produce a particular sensory event, only that we emphasize the produced event when calling this process *perception* and emphasize the way we produced it when calling it *action*.

The second general assumption acknowledges the neuroscientific insight that human brains do not code complex events in terms of separable symbols, but rather through distributed neural codes that are sensitive, and often selectively sensitive, to particular features of the event. For instance, the visual cortex is known to consist of a larger number of feature maps, which consist of neurons that code particular values of some common underlying dimension, such as the orientation, color, or motion direction of a given stimulus event (DeYoe & Van Essen, 1988). Feature-based representations have also been demonstrated for other sensory modalities (Saenz & Langers, 2014) and for movement parameters (i.e., perceivable and producible action features: Georgopoulos, 1990; Kalaska & Hyde, 1985), and thus seem to reflect a general characteristic of representations in the human (or primate) cortex (Von der Malsburg, 1999).

The third general assumption, which refers to earlier insights from Heider (1926/1959) and Brunswick (1944), derives from the conclusion that perceptual events and actions can be related to each other in terms of the distal events they refer to (e.g., the distance a hand should travel should fit with the perceived distance between the hand and a cup one intends to reach before starting the movement) but differ drastically with respect to their proximal codes (e.g., the neural firing pattern representing the distance in the visual system and the firing pattern representing the to-be-traveled distance in the motor cortex; Prinz, 1992). In other words, perception and action can effectively communicate only if their language refers to features of the external world but not to the features of the codes representing these external features. Accordingly, the common currency of representations involved in perception and action need to be distal features.

### Specific assumptions

The five more specific assumptions of TEC refer to: (a) the multimodal nature of event features, (b) the activation and integration of feature codes, (c) the attentional/intentional modulation of event coding, (d) the roles of event codes (event files), and (e) the architecture of event representations. The first assumption directly follows from distal coding. If what it refers to in the external world is more important for a feature code than how this external information is registered and proximally coded, it must not care about the modality or the source of a particular information – if it only co-varies with the represented state of affairs in the world. In representing the greenness of things in the world, we are likely to take the activation of neurons in the color map of our visual cortex into account but may also consider the particular emotional state that green things may invoke in us, the memories green things might retrieve, the Gibsonian affordance greenness might provide, and so forth. Hence, over time and experience every event reflecting the “greenish” portion of the light’s wavelength will become represented by all the internal activities that systematically accompany (i.e., are strongly correlated with) the exposure to green things. This implies that the feature code GREEN is a type of concept in the sense of Feldman Barrett (2017), as it can be considered an internal construction of some external fact rather than a one-on-one translation of some objectively existing fact into internal activity (a characterization that would apply to proximal representations; see Heider, 1926/1959).

The second assumption makes a distinction between the activation of a feature code, such as the increase of activation of the GREEN code when seeing an apple, and the integration of multiple activated codes, such as the code GREEN with other apple-related codes like ROUND, EDIBLE, and GRASPABLE. Logically, activation sets in before integration can take place, which has important implications for predicting the effects of one perceived or planned event on the perception or planning of other events. While feature codes are activated but not yet bound, they tend to prime all representations that include this particular feature code – for example, seeing something green facilitates saying “green,” and vice versa. Once a feature code is bound into a more integral event file (a network of feature codes), whether it be when perceiving or planning the production of an event, it interferes with the perception or planning of other, feature-overlapping events (Stoet & Hommel, 1999, 2002).

The third assumption considers the contribution of each feature code to the representation of an event. Codes of features on a dimension that is (assumed to be) relevant for the presently relevant task will have a stronger impact on representing an event then codes of features related to currently irrelevant dimensions – the intentional weighting principle (Memelink & Hommel, 2013). For instance, in a task in which the two possible stimuli or responses mainly differ in horizontal location, stimuli and responses are more likely to be coded in terms of left and right than in a task in which stimuli and responses differ on non-spatial dimensions.

The fourth assumption refers to the roles that feature or event files can play. In principle, each code can represent either stimuli or responses or both (the common coding principle). This means that stimulus and response codes do not differ in type or format, but only with respect to the role they play in a particular situation or task.

The fifth and final assumption refers to the architecture of event representations. Feature codes are assumed to be grounded in sensorimotor experience; this is where they come from and the process through which they emerge. This is true for features that are related to physical dimensions of external events, such as form and color, features related to one’s own body – such as kinesthetic and tactile feelings accompanying a movement, features related to actions – such as grasping or throwing (thus creating Gibsonian affordances like GRASPABLE and THROWABLE), and features related to the affective response one has to an event, given that these are merely interpretations of perceived interoceptive activity (Feldman Barrett, 2017; James, 1884). Given that each of these features might become associated with other features for reasons that are unrelated to the respective event (e.g., by learning that GREEN commonly implies FRESH), the experience of an event might be accompanied by the activation of features that are not directly given by the experienced event. Moreover, perceptual and action learning are likely to result in event representations that are more complex and more integrative than the one-shot event examples I have given so far.

## The Theory of Event Coding V2.0

Since its development in the late 1990s, TEC has inspired numerous studies on the interaction between perception and action. In contrast to earlier information-processing models of stimulus-response compatibility, TEC not only accounted for compatibility effects of various kinds but also allowed for the interpretation of phenomena like imitation and mimicry (Brass, Bekkering, Wohlschläger, & Prinz, 2000), effects of action planning on perception (Fagioli, Hommel, & Schubotz, 2007; Müsseler & Hommel, 1997), sequential effects of stimulus and response coding (Hommel, Proctor, & Vu, 2004; Spapé & Hommel, 2008), and interactions between concurrently planned actions (Stoet & Hommel, 1999). Some of these effects were successfully modeled in computational versions of TEC (Haazebroek, Raffone, & Hommel, 2017; Kachergis, Wyatte, O'Reilly, de Kleijn, & Hommel, 2014), neuroscientific studies have uncovered some of the neural underpinnings of TEC-related processes (Elsner et al., 2002; Kühn, Keizer, Colzato, Rombouts, & Hommel, 2011a; Kühn, Keizer, Rombouts, & Hommel, 2011b), and developmental studies have investigated the acquisition of TEC-related knowledge structures (for a brief review, see Verschoor & Hommel, 2017). In the following, I would like to review some of the most recent developments of TEC, as they expand the scope of the theory and their application significantly. Note that these new developments, and the theoretical additions they resulted in, are not meant to replace the original TEC 1.0, but rather to provide theoretical add-ons that specify or modulate the representations and mechanisms introduced by the original version. In other words, TEC 2.0 fully includes TEC 1.0, which is why the following discussion concentrates on the new features of TEC 2.0.

### The Control of Event Coding

Adopting the ideomotor idea that actions are integrated with and represented by codes of their perceptual effects – and assuming that these effects are represented in terms of feature codes – allows TEC to model the representations of actions in the same way as Kahneman, Treisman, and Gibbs (1992) have modeled the representations of perceived events. These authors have pointed out that the fact that perceptual events are represented by neural activity that is distributed across wide areas of the cortex requires some sort of integration. Indeed, the cortex of humans and other primates is known to code various features of perceived events in dedicated feature maps, such as color- and shape-specific maps in the visual cortex code and frequency-specific maps in the auditory cortex. In an environment that consists of more than one object at a time, the brain thus needs to determine which of the various neural activities belong to the same event – the notorious binding problem (Treisman, 1996). While there is no consensus with respect to the question of how the binding problem is neurally resolved, a large body of evidence suggests that people do bind features of the same event together. For instance, the facilitation of letter naming by presenting the to-be-named letter before is enhanced if the present and previous letter appeared as part of the same object (Kahneman et al., 1992). Along the same lines, repeating a visual, auditory, or tactile feature of an event facilitates performance on the repeated feature only if other features also repeat (Hommel, 1998) – otherwise facilitation turns into interference (the partial-repetition cost). Assuming that action plans are represented in the same way as are perceived events suggests that feature binding may not be restricted to classical perceptual features but also extend to action features (as according to TEC they are perceptual features of the same right and nature). Indeed, numerous studies have shown that partial-repetition costs can also be demonstrated for combinations of stimulus and response features (Hommel, 1998) and for combinations of action features belonging to different action plans (Stoet & Hommel, 1999). Hence, people bind features of perceived events, of intentionally performed actions, and across perception and action.

If feature binding serves the purpose of organizing neural activity belonging to different external events, one would expect that features are bound rather automatically, that is, irrespective of current goals and intentions and irrespective of the need or use of binding these features. The same conclusion is suggested by ideomotor theorizing, which implies that actions and their perceptual effects are bound spontaneously, and long before these bindings are used for intentional action planning (Verschoor & Hommel, 2017). However, first observations from feature-repetition-priming studies seemed to suggest a different picture. For instance, Hommel (1998) had participants carry out actions in response to either shape or color features. Repetitions of the task-relevant stimulus feature always strongly interacted with repetitions of the response: if shape was relevant, performance was better if both shape and response were repeated or if both alternated, as compared to the partial-repetition conditions (i.e., if shape was repeated and response alternated or vice versa), and if color was relevant, it was color and response repetition that strongly interacted. The task-irrelevant feature (color in the first and shape in the second example) also showed some interaction with response repetition, but that was always weaker and sometimes non-significant. These and related observations made me think that feature dimensions might be weighted according to their task-relevance, in such a way that features of task-relevant dimensions might induce a stronger activation than features of task-irrelevant dimensions, and that only features passing a particular integration threshold would become integrated into event files (Hommel, 2004). In other words, feature binding might be selective, and depending on task goals, more selective than feature-integration and ideomotor accounts seem to suggest.

However, considering the nature of feature-repetition-priming studies, this is not a necessary conclusion. If the repetition effects of two features interact, one needs to assume that (a) the codes of these two features were bound during or as a consequence of the previous presentation, and (b) the created binding is retrieved by re-viewing at least one of the integrated features. If no significant effect is obtained, however, or if that effect is weakened, this may be because no or less binding occurred on the first presentation, because no retrieval took place on the second, or both. In other words, a lesser or absent effect need not indicate lesser or absent feature *binding*, but may rather point to an effect on binding *retrieval*.

Interestingly, converging evidence points rather consistently to retrieval, rather than to binding, as the cause of selectivity. For instance, partial-repetition costs for task-relevant dimensions are less pronounced in individuals with high fluid intelligence (Colzato, van Wouwe, Lavender, & Hommel, 2006a) and in young adults, as compared to children and older adults (Hommel, Kray, & Lindenberger, 2011), and more pronounced in children with autistic spectrum disorder (ASD; Zmigrod, de Sonneville, Colzato, Swaab, & Hommel, 2013). If partial-repetition costs really reflect feature binding, this pattern is the opposite of what one would expect: if anything, subpopulations that have been suspected to have greater difficulties in integrating information (like individuals low in fluid intelligence, children and older adults, and patient suffering from ASD) should show smaller, rather than larger, binding effects. However, if one takes partial-repetition costs to indicate retrieval, the obtained pattern would make sense: Note that the task neither requires nor rewards the retrieval of previous bindings, suggesting that reduced effects indicate better control over information retrieval. Given that such control should indeed be more effective in individuals with high fluid intelligence and in healthy, young adults, the obtained outcomes would be more intuitive.

More direct evidence for a role of retrieval was provided by a study in which the task-relevance of stimulus features was systematically varied in time. As in typical feature-repetition studies (e.g., Hommel, 1998), participants were presented with pairs of stimulus-response combinations: they were cued to respond to the first stimulus (S1) by a particular response (R1), so that neither the shape nor the color of S1 mattered, and would then see another stimulus (S2), the shape or color of which would indicate the second response (R2). As usual, it was expected that repeating or alternating both stimulus shape and response and repeating alternating both stimulus color and response would yield better performance than repeating the stimulus feature while alternating the response, or vice versa (the partial-repetition cost). However, which feature dimension of S2 would indicate R2 (shape or color) was signaled either before or after the presentation of S1. If the dimensional cue appeared before the presentation of S1, the binding of S1 and R1 features would thus occur under the attentional set that this cue would indicate. If this binding is selective, the resulting partial-repetition costs should be stronger for repetitions of the cued feature dimension and the response than for the non-cued feature dimension and the response. If the dimensional cue appears after S1 presentation, however, S1-R1 binding should be under the influence of the attentional set that was relevant in the previous trial. Interestingly, the time point at which the dimensional cue was presented did not matter, suggesting that it is not the creation of bindings that is affected by the attentional set, but rather binding retrieval. This also fits results from two neurofeedback studies, which showed that training participants in increasing gamma-frequency-band activation of their frontal cortex reduces partial-repetition costs for task-irrelevant feature dimensions but not for the task-relevant feature dimension (Keizer, Verment, & Hommel, 2010b; Keizer, Verschoor, Verment, & Hommel, 2010a). The outcome pattern suggests that the training promoted the gamma-based top-down control of episodic memory by the frontal cortex, which in turn sharpened retrieval by increasing the focus to task-relevant features.

Taken altogether, the available evidence suggests that creating event files is an automatic process that binds features of perceived events, of intentional action plans, and of combinations thereof. However, people are able to selectively retrieve previously created event files when encountering one or more of the features they contain. The degree to which they can differ between individuals varies, but these differences can be reduced through training.

### The metacontrol of event coding

Traditional approaches to human action control still follow the pioneer of will psychology, Narziss Ach (1910, 1935), in trying to understand the nature of cognitive control by studying how and under which circumstances it manages to overcome overlearned habits. To that end, Ach had developed a method to experimentally induce particular habits by having participants carry out one kind of cognitive operation on a set of stimuli (Hommel, 2000), such as responding to nonsense syllables by producing a corresponding rhyming response (e.g., *zup*➔*tup*), for weeks before having them apply another operation, such as exchanging the first and second consonant (*zup*➔*puz*). As predicted, participants were slower and less accurate when carrying out the new operations on stimuli that were previously trained to generate responses that were no longer correct under the present instruction, as compared to stimuli from an untrained set. It is easy to recognize the same logic in currently more popular research designs investigating cognitive control, such as in studies using the Stroop or Simon effect, the only exception being that the interfering habit is no longer under experimental control but assumed to exist beforehand. According to the will-against-habit idea underlying this practice, cognitive control is active and successful to the degree that the previously acquired habit can be overcome. Hence, the smaller the Simon/Stroop effect, the stronger one’s will (also known as “cognitive control” or “executive functions”: e.g., Verbruggen, McLaren, & Chambers, 2014).

While this logic is shared by virtually all current accounts of cognitive control, it may be too simplistic to capture the true complexity and adaptivity of human behavior. As has been argued by Goschke (2003), behavior is unlikely to be adaptive when shielding the current goal against all possible challenges: Ignoring task-irrelevant information might be useful when trying to meet a paper deadline or persist with one’s diet, but it can be less useful when encountering a predator or warrior of a hostile tribe during one’s search for food. Hence, truly adaptive behavioral control needs to know when to persist (in pursuing one’s goal) and when to let go (and become flexible enough to adopt other goals). Recent neuroscientific insights suggest that this needed balance between persistence and flexibility emerges from the interaction between the mesofrontal and the nigrostriatal dopaminergic pathway (Cools & d’Esposito, 2010) and/or between receptor families residing therein (Durstewitz & Seamans, 2008).

Another complication stems from the fact that humans commonly entertain more than one goal: They may well try to be fast in a laboratory task, but they at the same time want to save energy for the upcoming seminar, keep in mind their shopping list for later, satisfy their economic needs, maintain their good manners and moral ideals, build their career, etc. It is unlikely that handing a student a small fee or credit point and instructing them to carry out what seems to be a rather nonsensical task will bring all these other goals to a halt. If so, behavioral control is likely to represent a useful compromise between all these goals and the information processing style they suggest. While many goals may not show any obvious bearing to a Stroop task, say, some are likely to directly impact performance on it. For instance, recent predictive-coding approaches (e.g., Pezzulo, Rigoli & Friston, 2018) have revitalized the idea that humans are constantly busy with predicting upcoming events and try to improve their predictions whenever they fail – a renaissance of the approaches of Berlyne (1949, 1960) and Sokolov (1963). If true, this suggests that people are particularly interested in uncertain stimuli, such as the commonly randomly chosen color words of Stroop stimuli. Thus while these words are not relevant and actually represent a hinderance for optimally performing the goal in the Stroop task, they should be highly relevant for a brain that is particularly interested in difficult-to-predict stimuli. Indeed, interference from nominally task-irrelevant stimulus features is drastically reduced or eliminated if these features become predictable (Frings, Merz, & Hommel, 2019).

These and other considerations (see Hommel, 2015b) suggest that adaptive control tries to find a balance between goal-shielding persistence and (less shielding or even goal-sacrificing) flexibility (Dreisbach & Goschke, 2004) by systematically biasing one’s style of information processing accordingly. Elsewhere (Hommel, 2015b; Hommel & Wiers, 2017) I have suggested that this is achieved by modulating (a) the degree to which the current goal(s) bias decision-making by supporting goal-compatible choice alternatives, and (b) the degree to which representations of such alternatives compete with each other. Under a persistence bias, the top-down support through goal representations and the mutual competition would be strong, but both would be weak under a flexibility bias. Among other things, this claim suggests the existence of interindividual and intraindividual differences in control style. Indeed, genetic predispositions that have been assumed to make the frontal persistence system more efficient improve performance on persistence-heavy tasks, such as Stroop-like conflict tasks, but not on flexibility-sensitive integration tasks, such as the attentional-blink task (Hommel & Colzato, 2017a), while predispositions supporting the efficiency of the striatal flexibility system have the opposite effect. Along the same lines, cultural factors propagating individualistic mindsets were shown to be associated with better performance in persistence-heavy tasks than factors propagating collectivistic mindsets, while the opposite was found for flexibility-heavy tasks (Hommel & Colzato, 2017a). Intraindividual differences were also obtained. For instance, meditation of the focused-attention kind (Lutz, Slagter, Dunne & Davidson, 2008) were found to improve persistence-heavy tasks, while meditation of the open-monitoring kind was reported to improve performance in flexibility-heavy tasks (Hommel & Colzato, 2017b).

To summarize, event coding takes place under a particular metacontrol mode, which can vary between extreme persistence and extreme flexibility. Persistence is characterized by a strong impact of the current goal and strong mutual competition between alternative decisions, while flexibility is characterized by a weak impact of the current goal and weak competition. As a consequence, a persistence bias facilitates discrimination between alternative events (as the underlying event files compete more in a winner-takes-all fashion) and cognitive/behavioral exploitation, while a flexibility bias facilitates integration and cognitive/behavioral exploration (Hommel, 2015b).

### Binding and learning

Event files can be considered episodic snapshots of particular feature combinations that are maintained over some time. The creation and maintenance of event files is thought to address the binding problem by organizing feature-based information in an event-specific way. But what happens if the event is over? Studies tapping into action-effect integration suggest that event files are maintained for at least the duration of an experimental session, and the theoretical assumption is that they are stored even longer (Elsner & Hommel, 2001). This raises the question of how binding and the short-term integration of feature codes are related to long-term memory. More specifically, Logan (1988) has suggested that the acquisition of cognitive skills proceeds by storing instances of episodic experiences, which raises the question of whether the concepts of event file and of instance are related or even identical. Interestingly, the available empirical evidence suggests that the answer is yes and no.

Colzato, Raffone, and Hommel (2006b) set up a study that directly contrasted the effects of binding and learning on performance. A feature-repetition design was used in which the shape and color of a visual stimulus repeated or alternated orthogonally. As in previous studies, repeating or alternating both features yielded better performance than repeating one but alternating the other – the partial-repetition cost. However, two of the four possible combinations of shape and color were much more frequent than the other two. This produced a main effect of frequency, so that performance was better for the more frequent combinations. Importantly, however, this effect did not interact with the partial-repetition cost, suggesting that binding was independent from learning. Further experiments with already overlearned feature combinations (like yellow bananas and red strawberries as compared to red bananas and yellow strawberries) showed the same independence, which rules out that it might reflect limited experience.

These findings were fully replicated in a later study by Hommel and Colzato (2009), who suggested a dual-process account: Repeated experience of particular feature combinations results in a long-term memory trace (perhaps a Loganian instance) that interacts with incoming perceptual information. If, thus, a banana is encountered, the activation of the corresponding shape code may directly prime the associated YELLOW code, which speeds up processing and responding to yellow bananas. Independent of this general benefit, however, the banana shape code will be just as effectively bound to a RED code if the currently perceived banana happens to be red, and it would be bound to the YELLOW code if the banana is yellow. Hence, the event file that serves for the online representation of the banana is not identical to the event file or instance that is kept in memory, even though reactivating the memory file might activate a code that, in the absence of conflicting perceptually-derived codes, might become bound to the online file. This difference between memory and online file might be related to different neural mechanisms. While longer-term storage is likely to rely on structural changes, online binding might rely on neural synchronization (Hommel & Colzato, 2009). It is certainly possible that bindings are transferred/transformed into memory traces (e.g., neural synchrony over some time or with some intensity might induce structural changes), but these traces operate independently from the bindings (cf., O’Reilly, Bhattacharyya, Howard, & Ketz, 2014). In other words, binding might lead to, but is not identical to, learning, which in turn implies that event files are not (just) but might become instances (or memory event files).

To summarize, there is evidence that online feature binding is not directly supported by stored knowledge, even though activated memory codes may well become integrated into online feature binding (as the fifth specific assumption of TEC 1.0 implies). At this point, it remains unclear whether and exactly how online feature bindings are transformed into more durable memory event files. In other words, more research on the relationship between binding and learning is wanted.

### The coding of social events

The original aim of TEC was to account for interactions between perception and action that unidirectional stage models were unable to explain. The situations in which such interactions were studied inside and outside the lab were commonly non-social in nature: some stimuli facilitated some actions more than others and some actions increased sensitivity to some stimuli more than to others. Even in situations that included other individuals, such as in imitation studies, the social aspect was considered to be irrelevant. However, more and more cognitive researchers became interested in social situations and asked whether the presence and characteristics of another person, the actions the person is performing, and the goals he or she is pursuing might have an impact on cognitive and behavioral performance. For instance, Knoblich and Sebanz (2006) claimed that perception and action are inherently social in nature and that other people’s actions and intentions are automatically included in one’s own situational representation. This in turn raises the question of how people cognitively represent other individuals and how they distinguish between themselves and others (e.g., when taking turns in a cooperative task).

On the one hand, one might find that TEC is simply not made to account for social effects and leave the theorizing to dedicatedly social frameworks – and this was, indeed, my first reaction to the first more interactive studies. On the other hand, however, it is difficult to see why a theory that aims to explain how events are coded should surrender only because a given event is created by another person and/or involves characteristics that refer to living beings rather than static objects. Indeed, the ideomotor underpinnings of TEC already include the aspect of self-perception, which means that the action-related representational assumptions of TEC are already tapping into aspects of self-representation. These were the reasons to explore whether and how TEC could be extended to include the coding of social events.

According to Greenwald et al. (2002), concepts of the self can be considered networks of codes that represent the relevant features that what has been learned possesses. Some of these features might refer to physical characteristics, such as the size of one’s body, the color of one’s hair, or the speed of one’s gait, but others are likely to refer to more abstract characteristics, such as having a particular gender and social status, being a parent and supervisor, having a particular degree of intelligence, having particular preferences, etc. TEC can easily account for the physical features, as coding the size of a human body should not be any different from coding the size of a non-living object. However, how about more complex features?

On the one hand, it is clear that the complexity of a concept like parenthood requires more than registering a particular core feature in primary visual cortex. But that also accounts for concepts related to non-living objects, such as a table or university. Such concepts rely on more than one feature or a specific feature combination, i.e., a loosely defined set of features and feature combinations that can vary from instance to instance. On the other hand, however, there is no reason why such loosely defined feature sets should not be acquired through sensorimotor experience and eventually become ingredients of a continuously growing event-coding system (Hommel, 2016, 2018): For example, parenthood may be defined by experiences with other adults that play with and care for a particular infant, feeding and driving a particular adolescent to school, and hosting her on important holidays later on. Hence, the acquisition of more complex features is likely to take more time and to consider more information than the acquisition of simpler features, but there is no theoretical reason why the format of representing simple and complex features should differ and why social features should be coded any differently from nonsocial features. Once “social” features become coded into a person’s representation, encountering the person, or any cue related to this person, might reactivate the corresponding feature code stored in longer-term memory-based event files, and this reactivation will allow the feature to become part of an online event file. For instance, if a particular person has been known to show aggressive, attacking behavior in the past, encountering the person is likely to reactivate aggression-related codes even if such behavior is currently not shown, thereby preparing the perceiver to deal with the possible occurrence of this behavior.

### The coding of self

TEC does not distinguish between representing oneself and other individuals, so that representing oneself basically follows the same scenario as representing someone else. However, as discussed elsewhere (Hommel, 2018), the fact that a given perceiver-actor has more direct access to more information than he or she has about others introduces three important differences between self- and other-representation.

First, perceiving oneself and others is likely to be more similar with respect to information collected through exteroceptive perceptual channels, like vision, audition, tactition, and olfaction, than with respect to information collected through interoceptive channels, like proprioception and affective states. This is likely to make self-related representations more comprehensive and more strongly colored by codes derived from interoception, including affect.

Second, given that event files can also include (codes that can trigger) motor activity, the representation of one’s own actions is likely to be richer and more “motoric” than the representation of another person’s actions. However, this discrepancy depends on the degree to which self and other are sharing event-specific action information: if, say, an experienced ballet dancer is observing another ballet dancer, the perceptual input is likely to reactivate the same interoceptive and motor codes that the observed dancer uses to code her own action.

Third, the sensory consequences of one’s own actions are commonly easier to predict than the consequences of other people’s actions, again suggesting that representing oneself is likely to be based on richer information and more accurate predictions than representing someone else. However, this discrepancy again depends on self-other overlap and the degree of shared history; it will thus be much smaller in the case of the two experienced ballet dancers than in the case of two strangers meeting for the first time, especially if they come from different cultural backgrounds.

### Summary of novel contributions of TEC 2.0

As already mentioned, TEC 2.0 leaves the original 1.0 version fully intact but provides a number of important specifications and extensions that strongly enhance the scope of the theory. First, it distinguishes between the control of the actual binding process – that is, the integration of currently available and memory-based, reactivated features into online event files – and the control of the retrieval or reactivation of a just-created online event file. The available evidence suggests that the binding process is rather non-selective with respect to current goals, and thus integrates all feature codes that are currently activated, for whatever reason. In contrast, the retrieval or reactivation process seems to be very selective in restricting retrieval/reactivation to codes that currently are or that the individual believes to be task-relevant. Second, the handling and especially the retrieval/reactivation of event files is governed by current metacontrol states, which can vary from extreme persistence (implying high selectivity and focus) to extreme flexibility (implying the opposite). Third, online bindings and memorized networks of feature codes can be shown to have separable effects, but it remains unclear whether and how online feature bindings are transformed into more durable event representations. Fourth, social and non-social events are claimed to be coded the same way, by means of the same representational logic. This also holds for the representation of oneself, which, however, may often be richer than the representations of others.

## Integration and application

We have seen that the original TEC has been extended in various directions by adding assumptions about how simple and complex event files are created, handled, and retrieved, and transformed into more enduring traces. In the following, I attempt to make these extensions more transparent by applying them to variations of the Simon task and the effects these variations generate, starting with the classical task and ending with the joint Simon task that is carried out by two individuals. I also try to show how the extended TEC can account for observations that might look very confusing and divergent at first sight and that are difficult to understand from other theoretical positions.

### The Simon effect

**Fig. 1.**
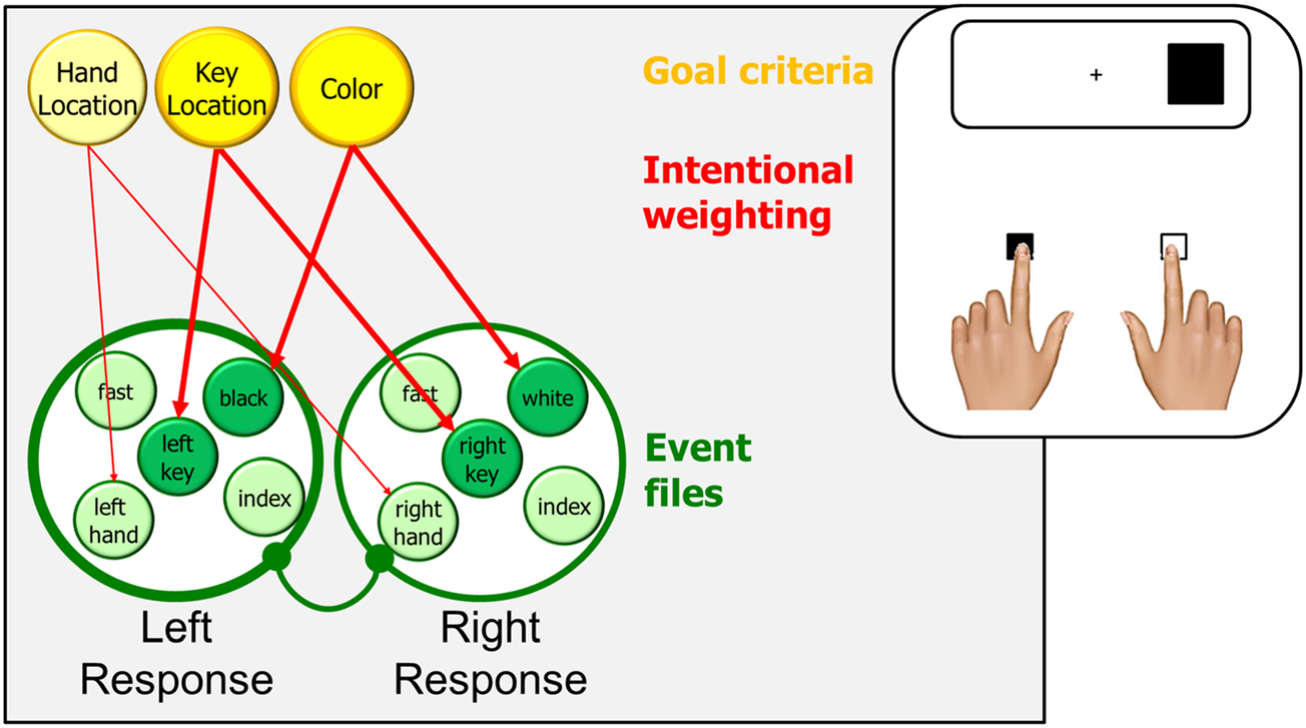
Representing left and right response events in a typical Simon task. Black and white squares are assigned to left and right key presses, respectively. Presenting the black square will activate the event file for the left response, which eventually is carried out. The instruction is couched in terms of key location, which renders this feature task-relevant in which increases the intentional weighting for key-location features. Hand location and other features are rendered irrelevant and are not or are only weakly weighted (shown only for hand location). Coding the location of the stimulus as RIGHT will activate the corresponding feature codes (right hand and right key in the example), which will spread to the other feature codes of the same event file to the degree that their intentional weight is sufficiently high. As a consequence, this event file will be a stronger competitor in response selection and, in the given example, slow down the selection of the left response or even result in performing the (incorrect) right response

The effect first reported by Simon and Rudell (1967) is widely used in the cognitive sciences because of its simplicity and its interesting theoretical implications (Hommel, 2011). The basic setup needed to obtain a Simon effect consists of two stimuli that randomly appear on the left and right side of some reference point, such as a fixation cross in the middle of a screen, and two responses that are defined by their horizontal location. While stimulus location is instructed to be task-irrelevant, a non-spatial feature of the stimulus, such as color or shape, signals correct responding. In the example given in Fig. 1, participants respond to black stimuli by pressing a left key and to white stimuli by pressing a right key. Notwithstanding the task-irrelevance of stimulus location, responses are commonly found to be faster and more accurate if the stimulus happens to spatially correspond to the response key (the compatible or congruent condition) than if the stimulus appears on the side of the currently incorrect response key (the incompatible or incongruent condition) – the Simon effect.

TEC accounts for this effect by assuming that stimuli activate the task-specific event files to the degree that they feature-overlap with them. As shown in Fig. 1, the left response in our example task might be represented by feature codes referring to the left hand that is being used, the index finger that is being moved, the left key that is being pressed, the high speed of the response, and the black color of the relevant stimulus (more features are likely to be coded but not essential for the explanation). The left response also contains the motor pattern needed to trigger the left response (not shown in the example), so that a sufficient activation of the left-response event file will lead to the execution of the left response. Along these lines, the right response might be represented by feature codes referring to the right hand, the index finger, the speed of the response, and the white color of the stimulus. If the black stimulus now appears on the right side of the screen, it will activate the event file of the left response by activating the black feature code that is bound to the representation of the left response. However, it will also activate the event file of the right response by activating the LEFT codes referring to the right hand and the right key, which in turn creates a response conflict that needs more time to resolve. This would not be the case if the black stimulus appeared on the left side, because it would then feature-overlap with the representation of the left response only and thus not create any response conflict.

While a pure event-file approach would suffice to account for the basic Simon effect, it fails to account for some of its variations. For instance, a Simon effect is also be observed if the left and right key are operated with two fingers of the same hand (Heister, Ehrenstein & Schroeder-Heister, 1986) or, as in crossed-arm studies, operated by the index fingers of the contra-lateral hand (Wallace, 1971). This suggests that the left and right response is mainly or exclusively coded in terms of the location of the key being pressed but not of the effector being used. To account for effects of that sort, TEC assumes that some features representing a particular event can receive a higher weight than others, either because their salience suggests so or because the respective feature dimension is directly or indirectly task-irrelevant. In the Simon task, the most salient difference between left and right responses is the location of the key being pressed (which, in the standard setup, is perfectly confounded with the location and the anatomical status of the active effector) and this location is also commonly used to refer to the responses in the instruction. This renders key location more task-relevant than hand location, say, which is indicated in Fig. 1 by the stronger coloring of the key-related goal criterion. Task-relevance leads to the higher intentional weighting of the corresponding features, as also indicated in the figure. As a consequence, the correspondence between the task stimulus and the key location will matter more than correspondence between stimulus and hand or finger location, which accounts for the available findings from crossed-arm studies.

### The inverted Simon effect

**Fig. 2.**
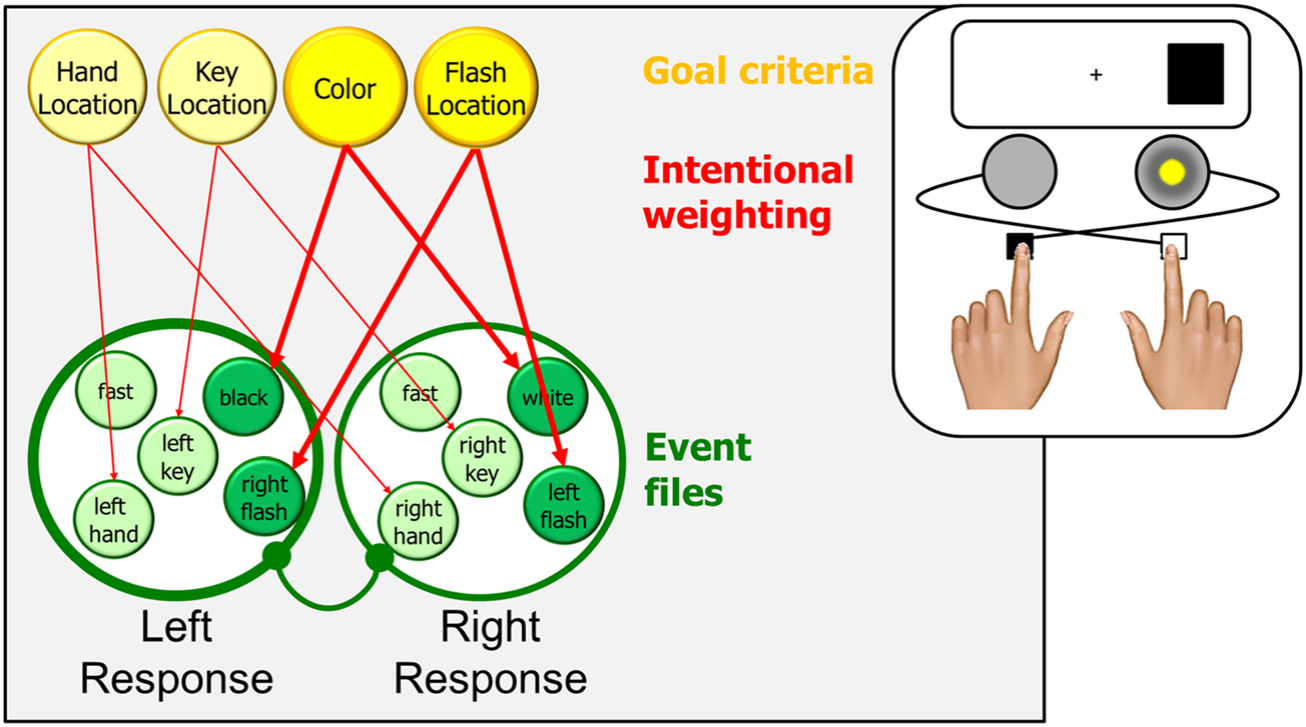
The processes leading to the inversion of the Simon effect. Left and right response events are basically coded as shown in Fig. 1, with two exceptions. First, the fact that the left response triggers a right light flash and the right response triggers a left light flash is represented by including the corresponding flash-location codes in the respective event files. Second, the fact that participants are instructed in terms of flash location rather than key location implies a change in goal criteria and a change in intentional weighting, which now increases the impact of flash-location codes and reduces the impact of key-location or hand-location codes. Coding the location of the stimulus as RIGHT will activate the flash-location (right flash in the example), which will spread to the other feature codes of the same event file. As a consequence, this event file will be a stronger competitor and response selection and, in the given example, speed up the selection of the left response

An even more important role of goal criterion and intentional weighting was demonstrated by Hommel (1993). This study actually used high- and low-pitched tones instead of black and white colors, but here I translate tones into colors for the sake of consistency. In one condition, pressing a left key would flash a light on the right side, while a right key would flash of light on the left side, as indicated in Fig. 2. This setup allowed instructing one group of participants in terms of key location (e.g., “press the left key if the black stimulus appears”) and another group in terms of flash location (“flash the right light if the black stimulus appears”). With key instruction, the goal criterion should be defined in terms of key location, so that (in addition to the impact of its color) a stimulus appearing on the left should mainly activated the left response and the stimulus appearing on the right the right response, thus producing a standard Simon effect. With flash instruction, however, the goal criterion should be in terms of flash location, as indicated in the Fig. 2. As a consequence, a left stimulus should mainly activate the event file representing the right response while a right stimulus should activate the file representing the left response. In other words, the Simon effect should be inverted with this instruction, which is exactly what was found.

### The Go-Nogo Simon effect

**Fig. 3.**
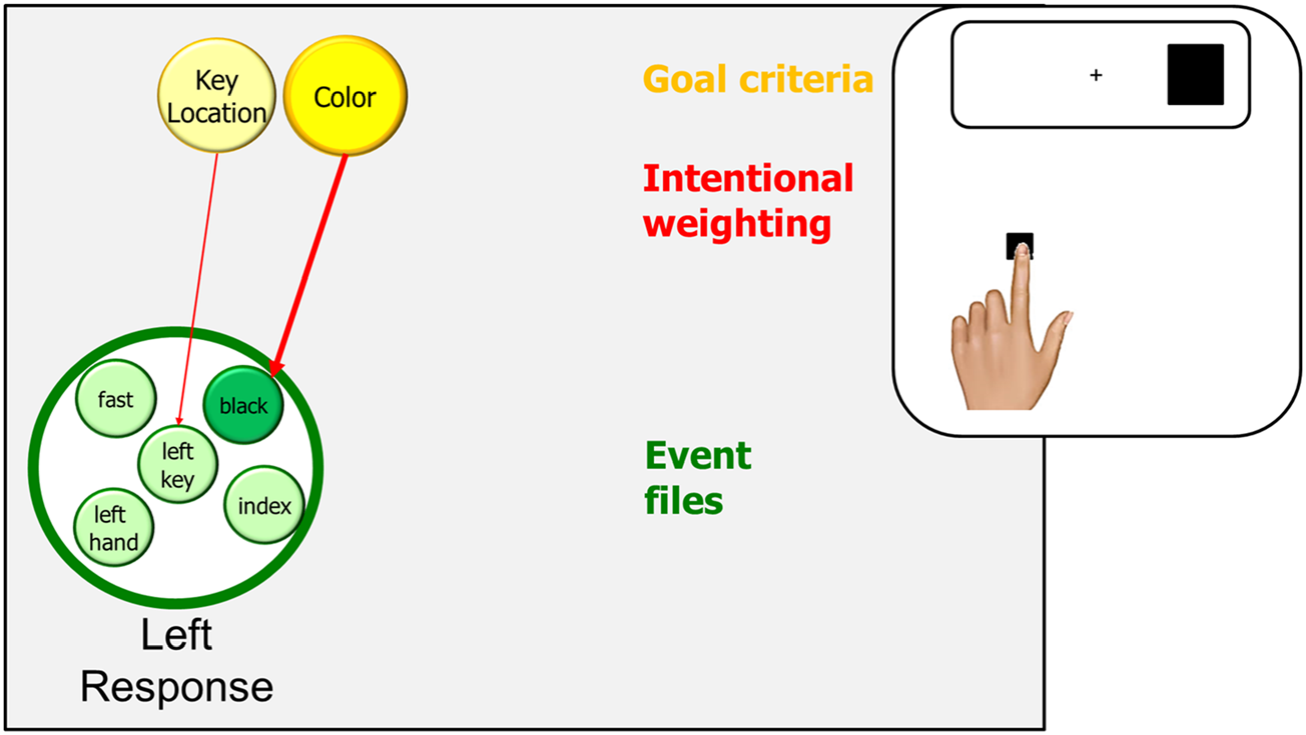
The (left in this case) response in a Go/Nogo Simon task. The participant responds to black stimuli and ignores white stimuli. There is no response competition and given that there is only one response, key location is not task-relevant. Stimulus location will be coded but given the task-irrelevance of all location-related feature codes, it will not activate ingredients of the event file to a sufficient degree to have a strong impact on response selection. As a consequence, no Simon effect will appear. However, frequently switching between left and right responses introduces a discrimination problem that would call for some degree of activation of the key-location code or similar codes that allowed to discriminate between the two responses. As a consequence, a small Simon effect will appear

The main contribution of processing-stage approaches to understanding the Simon and related stimulus-response compatibility effects consisted in the localization of the problem that these effects are thought to reflect in the processing chain. According to these approaches, response selection is the culprit: incompatible stimuli are thought to automatically activate the incorrect response, which results in response conflict that takes time to resolve (e.g., Kornblum et al., 1990). While TEC comes to the same conclusion, stage models did not provide any insight into why the automatic activation might occur but merely took this process for granted. Moreover, stage models face difficulties if it comes to accounting for findings obtained with Go-Nogo versions of the Simon task. Such a version is obtained by eliminating one of the two responses, for example by instructing participants to press the left key whenever a black stimulus appears. From a stage perspective, this would eliminate the need to select the response, so that the Simon effect should disappear (i.e., the left response should be as fast and accurate for left as for right stimuli). Indeed, this is what has been obtained in various studies (for a review, see Hommel, 1996), especially if response location was blocked over many trials. However, a significant Simon effect has been reported from studies in which participants often switch between left and right responses (Hommel, 1995, 1996). As such switches were always predictable and allowed for sufficient preparation, stage models are unable to account for the obtained effects. In contrast, TEC suggests that the presence of an alternative event in the same or a similar context introduces the need to distinguish between the available events. Given that location (be it hand, finger, or key location) is the most obvious discriminative feature between left and right response events, it makes sense to assume that the corresponding feature code, such as the key-location code in the example, is active to the degree that discrimination is necessary. As this should be the case as the frequency of switching increases and, thus, the recency of the alternative event decreases, frequent switching should indeed introduce at least a small Simon effect (Fig. 3).

### The joint Simon effect

Recent years have seen a steadily increasing interest in situations that involve more than a single individual working in a dedicated lab, and the joint Simon task has enjoyed particular attention in developing research designs consisting of multiple individuals working on the same task. Sebanz, Knoblich, and Prinz (2003) were the first to have two participants share the same Simon task. Distributing the task over to people turns the original binary-choice task into a Go-Nogo task: one participant operates the left key in response to one stimulus feature (black in my example) and the other participant operates the right key in response to another feature (white, for example). From a processing-stage point of view, the presence of the other individual should not matter, as the correct response could still be selected and prepared in advance. Hence, no Simon effect is predicted but, interestingly, significant effects are obtained when two individuals work side by side (Sebanz et al., 2003). How is that possible?

**Fig. 4.**
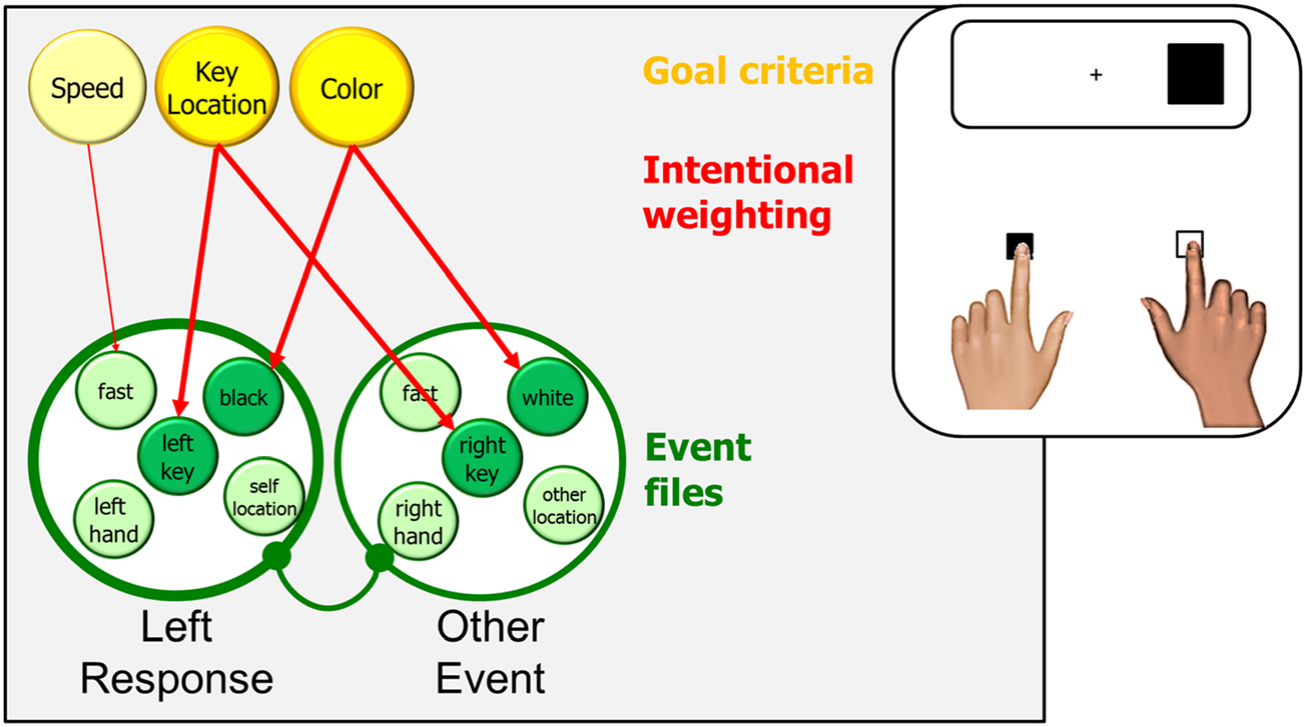
The (left in this case) response in a Joint Simon task (where the right key is operated by another person). The participant responds to black stimuli and ignores white stimuli. The presence of another event (here, the responses of the other individual) makes it necessary to distinguish between one’s own responses and this other event. Key location (or self-location which is confounded here) are likely to serve as the most salient distinguishing feature dimension, and so the key-location feature receives high intentional weighting. Accordingly, response competition is reintroduced and left stimuli will facilitate and right stimuli interfere with selecting the correct response

Various authors have offered the idea that participants might co-represent the task of the other person in an automatic fashion, so that the cognitive representation of the joint task would not differ much from the representation of the solo Simon task (e.g., Knoblich & Sebanz, 2006). However, such accounts have been shown to run into various difficulties, as indicated by evidence that the joint Simon effect can be obtained even if the other participant is replaced by a waving cat or a ticking metronome (Dolk, Hommel, Prinz, & Liepelt, 2013) and that several important aspects of the other’s task are not considered by the participant (Yamaguchi, Wall, & Hommel, 2018). However, if we consider the above-mentioned evidence that fast switching between alternative events is sufficient to reintroduce a Simon effect even in a Go-Nogo task and combine that with the TEC assumption that actions are represented by their perceptual effects, a non-social interpretation presents itself. As discussed in the previous section, having perceived oneself as carrying out an alternative action event just recently seems to introduce the need to discriminate that event from the event one is expected to generate in the present trial(s), with key/finger location being the most salient feature dimension for the purpose. Given that TEC assumes that self-produced and other-produced events do not differ in representational format (but only in the amount of sensory information and the predictability thereof; Hommel, 2018), it should not matter much whether one was producing the alternative event oneself or whether one observed someone else doing that. Accordingly, an obvious account for the joint Simon effect is that the recent experience of some other-produced alternative event (irrespective of whether that is produced by a person or a mechanical agent) has the same consequences as the experience of producing such an event oneself – the higher intentional weighting of key/finger location (Fig. 4), which in turn produces a significant Simon effect (Dolk et al., 2011).

### Modulation of the joint Simon effect

The non-social TEC account of the joint Simon effect assumes that the difficulty in discriminating between one’s own action event in a Go-Nogo task and another event leads the agent to emphasize the discriminative spatial feature of his/her response, which in turn increases the Simon effect. The difficulty to discriminate should increase with the saliency of the other event, which fits with the already mentioned observations that the joint Simon effect can be induced by non-social sources of difficult-to-ignore auditory stimulation (Dolk et al., 2013). However, there are also observations that the joint Simon effect is modulated by factors that are unrelated to objective saliency.

**Fig. 5.**
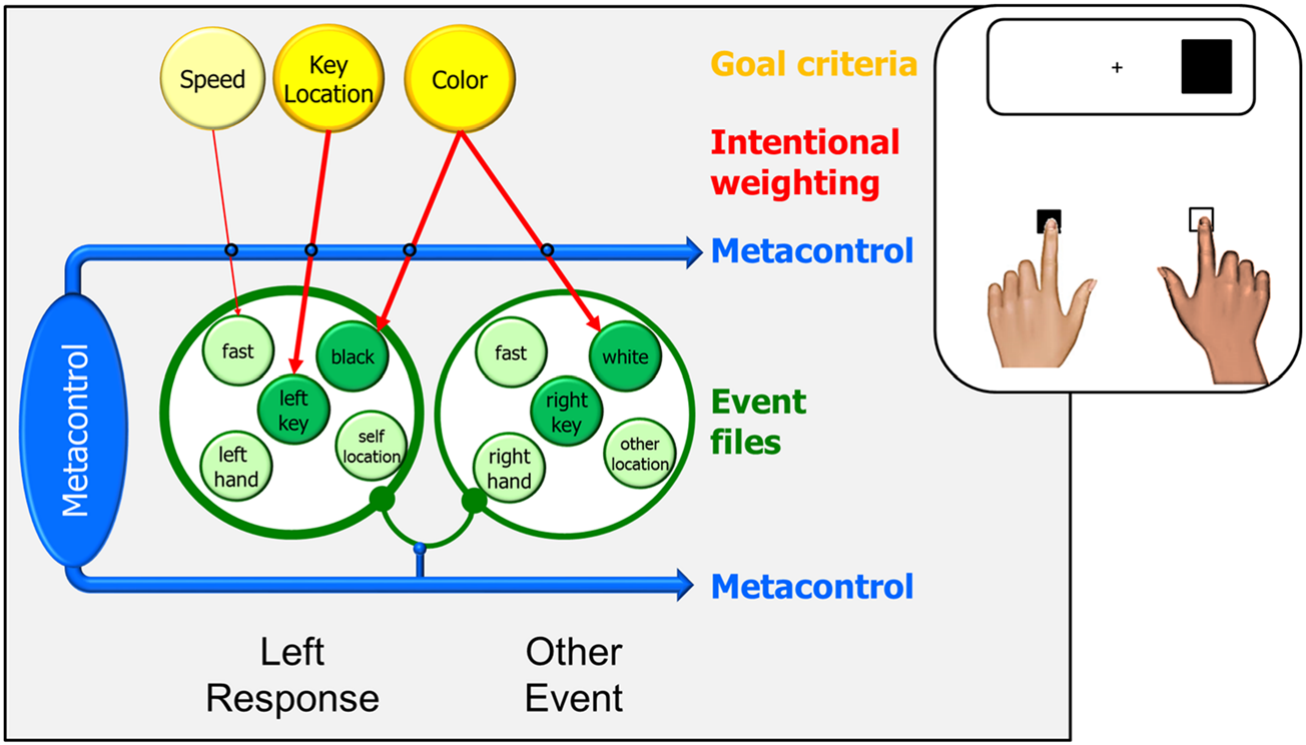
The impact of culture, religion, and mindset on the Joint Simon effect. Metacontrol modulates the impact of goal criteria on intentional weighting and the degree of mutual competition between event files. Individualistic attitudes increase, and collectivistic attitudes decrease impact and competition. Less competition increases perceived self-other overlap and, as a consequence, introduces a decision problem: which event is the correct response? This in turn renders event-discriminating features task-relevant, with key (or self-) location being the most obvious choice. As a consequence, key location receives higher intentional weighting, which in turn increases the joint Simon effect

For example, Colzato et al. (2012a) compared Taiwanese practicing Buddhists with Taiwanese atheists in a joint Simon task. The rationale was motivated by Buddha’s claim that practicing Buddhist meditation leads one to reduce and eventually eliminate the perceived borders between self and other (Harvey, 2012). If that were true, Buddhist participants should be facing a greater difficulty in discriminating between their own action and that of another in a joint Simon tasks, which in turn should increase the joint Simon effect as compared to atheists. This was indeed the outcome, suggesting that the same degree of objective saliency can induce different degrees of response conflict in people who differ in faith and lifestyle. In another example, Colzato, de Bruijn, and Hommel (2012b) had participants to work through tasks drawing attention to either personal interdependence or personal independence (Kühnen & Oyserman, 2002) before working on a joint Simon task. As expected, interdependence priming resulted in a more pronounced joint Simon effect, again suggesting that objective saliency of the alternative event is not the only factor that determines discrimination difficulty (Fig. 5).

At first sight, the finding that two factors with a very strong social flavor (faith/lifestyle and interdependence priming) increase the size of the joint Simon effect seems to challenge the above-mentioned non-social account. Indeed, the mechanisms discussed so far are insufficient to explain how such factors could operate, which brings us to metacontrol. As explained earlier, metacontrol states vary between persistence and flexibility, and they do so within and between individuals. As discussed, people with individualistic mindsets have been shown to excel in persistence-heavy tasks while people with collectivistic mindsets excel in tasks drawing on flexibility. This suggests that Buddhists, who are actively engaged in increasing the collectivistic nature of their mindset, are likely to possess a default metacontrol state that is more biased towards flexibility than the metacontrol state of non-Buddhists. If so, the Buddhist participants of Colzato et al. (2012a) can be expected to have more overlap between the representation of themselves and the representation of their co-actor, which should have increased the discrimination between their own response and that of the co-actor. This, in turn, should have made them rely more on the discriminating spatial feature of the response than atheists, so that a stronger joint Simon effect in Buddhists was indeed to be expected. The same goes for interdependence priming: If interdependence priming makes people place stronger weights on features they share with others, self-other overlap should have been increased and, as a consequence, the discriminating spatial feature should have been emphasized more.

Note that this last interpretation raises an interesting issue. In a nutshell, I have suggested that Buddhist and other practices aiming at a more collectivistic mindset bias metacontrol states away from persistence and more towards flexibility. Extreme flexibility is assumed to reduce competition between alternative codes and, thus, reduce perceptual and representational discrimination. Should this not also reduce spatial discrimination, which in turn would be expected to reduce, rather than increase, the joint Simon effect? In principle, this is indeed what the theory would predict, were it not that this would eliminate the only remaining distinction that the highly flexible participant can rely on to solve the task. This in turn introduces the concept of *task-constraints*, which we investigated in a recent study (Mekern, Sjoerds, & Hommel, 2019). The original idea was that individuals might systematically differ with respect to their personal biases towards persistence and flexibility. To reveal such biases, we had participants perform various tasks that all provided separable measures for the relative degree of persistence and flexibility (e.g., the number of trials in which an individual would persist in using the same letter set in a Scrabble task before requesting a new set). If personal biases were consistent, one would expect the persistence/flexibility measures to correlate across tasks, which, however, was not observed (except for tasks that were almost identical). Rather, what correlated across tasks was absolute performance, showing that individuals who excelled in one task were also likely to be good in another. My colleagues and I concluded that the particular tasks being used put such strong constraints on relative persistence and flexibility that participants could not afford to express the biases that were previously demonstrated in tasks with arguably fewer constraints (cf., Hommel & Colzato, 2017a). The correlations therefore did not reflect such biases, but, rather, the adaptivity of participants in adjusting metacontrol to the task requirements. Along these lines, I speculate that our Buddhist participants would indeed show reduced spatial discrimination in tasks that do not necessarily require such discrimination, but failed to do so in a joint Simon task because the task constraints do not only require some degree of discrimination (so to solve the task at all), but also strongly favor spatial over other kinds of discrimination. Clearly, more research is needed to test these speculations and, given their particularly salient spatial nature, Simon-like tasks will be unlikely to be the most suitable tool to provide such a test.

### Desiderata

The original TEC has received broad support from behavioral, neuroscientific, and modelling studies, and stimulated various new research lines. The extension of TEC in terms of control functions and representational scope has further increased its applicability, which now includes complex tasks and social, perhaps even sociological, phenomena. And yet, much more work needs to be done to fully exploit the theory’s potential. For one, it seems important and useful to better understand diagnosing metacontrol states. As long as instruments to diagnose such states in terms of relative persistence or flexibility independent of the performance characteristics that these states are thought to explain are not available, the danger of circular explanation remains. A first step would be to use different tasks for diagnosing the current state and for testing its impact, but even more independent behavioral indicators, neurophysiological correlates, or model-derived parameters would be extremely useful. For another, reconstructing more and more social phenomena in order to reveal their cognitive underpinnings would make it easier to apply TEC to them. This will often require stripping social-psychological descriptions of these phenomena from their phenomenological overhead. For instance, while dozens of “cognitively rich” definitions of trust or conformity exist, it seems possible to reduce their essence to predictability and self-other overlap (Hommel & Colzato, 2015; Kim & Hommel, 2015), which allows for various TEC-based predictions that are likely to stimulate novel research lines.
